# Decreasing reproductive and abortion care barriers: findings on the student health center's role from a student survey

**DOI:** 10.1186/s12905-023-02230-5

**Published:** 2023-02-24

**Authors:** Cynthia D. Rohrer, Sepideh Modrek

**Affiliations:** 1 Public Health Department, San Francisco State University, 1600 Holloway Avenue, San Francisco, CA 94132 USA; 2 Health Equity Institute and Department of Economics, San Francisco State University, 1600 Holloway Avenue, San Francisco, CA 94132 USA

**Keywords:** Reproductive health, Abortion care, Health equity, Barriers to access, Stigma, Medication abortion, Student health centers

## Abstract

**Background:**

College-aged young adults in the US have low utilization and high need for reproductive healthcare. Multiple barriers to reproductive care exist. University Student Health Centers (SHCs) provide varying degrees of reproductive products and services. Recently, California legislated that public university SHCs add medication abortion to their care.

**Methods:**

To examine existing attitudes and barriers to reproductive healthcare for public university students, we conducted an anonymous online survey at a large, diverse, urban coastal California State University. Students were asked about numerous barriers accessing reproductive services in general and at the SHC, which we categorized into three groups: stigma, access and system. Respondents were also asked about knowledge and preferences for accessing and recommending various services. To understand the extent to which inequities exist, we compared differences across racialized/ethnic identity, gender identity, anticipated degree, and living distance from campus using chi-squared tests.

**Results:**

The majority of survey (n = 273) respondents experienced stigma and access barriers in general healthcare settings which made obtaining reproductive healthcare for themselves or their partners difficult (stigma barriers 55%; 95% CI 49%–61%; access barriers 68%; 95% CI 62–73%). Notably, students reported statistically significant lower rates of access barriers at the SHC, 50%, than in general reproductive healthcare settings, 68%. There were limited differences by student demographics. Students also reported a high willingness to use or recommend the SHC for pregnancy tests (73%; 95% CI 67–78%), emergency contraception pills (72%; 95% CI 66–78%) and medication abortion (60%; 95% CI 54–66%). Students were less likely to know where to access medication abortion compared to other services, suggesting unmet need.

**Conclusions:**

Our study provides evidence that students face barriers accessing reproductive healthcare and that SHCs are a trusted and accessible source of this care. SHCs have a key role in increasing health, academic and gender equity in the post-Roe era. Attention and financial support must be paid to SHCs to ensure success as state legislatures mandate them to expand reproductive and abortion care access.

## Background

College-age young adults (18–24) in the US have low utilization rates of reproductive and sexual health services along with the highest rates of sexually transmitted infections (STIs), unintended pregnancy, and abortion [1–3]. Young adults simultaneously have less experience navigating sexual and reproductive health services and are in the peak age group for behaviors associated with the aforementioned sexual and reproductive health outcomes including unprotected sex, having multiple sex partners, and binge substance use [4]. Research shows most college students (89%) report their education goals would be negatively impacted by having a child, and women who do have a child during college have diminished graduation rates [3]. Two frequently cited reasons for having an abortion are financial concerns and disruption to education [6]. Thus, reproductive health and abortion care are essential services for student academic success, gender equity, and reproductive autonomy [3, 5, 7, 8].

College students are a sizeable proportion of the population and increasingly reflective of the national demographics (40% over age 25, 44% BIPOC, 34% first generation) with significant health needs despite their misperception as “privileged, resourced, and healthy”[2] p16] The global COVID-19 pandemic has worsened health determinants including housing, financial, and food insecurity, all of which exacerbate inequalities for BIPOC and low socioeconomic status (SES) students [2]. BIPOC and low SES populations, including students, also experience disproportionate inequities in sexual and reproductive health access, care, and outcomes [3, 5, 8]. Colleges are thus a critical setting for public health equity intervention with the potential to impact the reproductive health of women and people who can become pregnant, students of color, and low SES students [5, 9]. The majority of college or university student health centers (SHCs) nationwide provide some sexual and reproductive health services, which may include abortion counseling or referral, and Healthy Campus 2020 includes multiple objectives to improve student sexual and reproductive health [4, 10]. As disparities to access increase nationally, research indicates SHCs can reduce critical determinants of reproductive and abortion care access such as travel distance, facilitate retention and graduation, and impact rural and other college students who face a limited and decreasing number of providers [3, 11–14].

Previous research shows that knowledge of services and stigma experiences impact student access to reproductive and sexual healthcare on campus [1, 12]. Understanding the barriers to these services is critical as some state legislatures turn to SHCs to broaden access to reproductive healthcare generally, and to abortion care in particular, by mandating the provision of medication abortion care on public university campuses. In 2019 in California, these barriers persuaded the California legislature to enact the nation’s first law that required public university health centers to offer medication abortion (SB-24: public university student health centers: abortion by medication techniques). In 2023 California will be the first state to implement such a law for the over 700,000 students at its 32 public universities, while other states including Massachusetts and New York have proposed similar legislation [5, 15]. Research informing such legislation focused on the decreased travel, scheduling, and cost burdens to students seeking abortion potentiated by providing this essential care at SHCs [3, 5, 7]. Our research contributes further by quantifying these and additional barriers a sample of diverse urban students face in accessing reproductive care. It is unique in assessing whether and which types of reproductive health barriers exist and how they differ in the community healthcare system versus on campus. While data on projected student medication abortion rates, reproductive health barriers, and SHC staff perspectives exist, we sought to document student voices, experiences, and needs [1, 3, 7, 14].

We conducted a student survey and asked students if the SHC is a known, trusted, and convenient setting for reproductive and medication abortion care. We asked if students knew of a place they could access different reproductive and medication abortion services in their community; and then quantified if they would use, have their partner(s) use, or recommend a friend use the SHC for those services. We further quantified if, where, and how they experience barriers to care. We anticipated differences by service location and demographic subgroups, so we examined results across racialized/ethnic identity, gender identity, anticipated degree (undergraduate versus graduate), and living distance from campus; at the locations of general reproductive care and the SHC.

## Methods

We invited students to respond to an anonymous online survey to understand students’ self-reported use of and attitudes toward reproductive and abortion services. We conducted this survey in anticipation of legislated medication abortion provision at California public university campuses starting in 2023.

### Recruitment

The study population was students at a minority-serving institution (MSI) public university in a large coastal Northern California city consisting of a diverse, majority-commuter student population, where 60.13% of undergraduate students receive federal aid and 42.58% receive Pell Grant funding [16]. Recruitment for the anonymous web-based quantitative survey was done via on-campus and virtual methods to reflect hybrid learning modalities  at the university due to the COVID-19 pandemic. Eligible respondents were self-reported students aged 18 and over. We posted and distributed recruitment flyers with QR codes on campus at residence halls, academic buildings, the library, wellness (athletic) center, SHC, food court, and Student Union. We used college listserver emails inviting students to participate in a “Reproductive Health and Access Study” via a live link. We posted the survey announcement in the online student life hub calendar. Survey data was collected on the Qualtrics platform from March 01, 2022–May 02, 2022.

### Measures

The distributed survey contained 9 modules, with response-based skip patterns determining the survey length for each participant. Most participants spent between six to eleven minutes responding to the survey. Measures included demographic characteristics (gender identity, age, race/ethnicity, learning modality, anticipated degree, employment status, insurance status, housing situation, distance from campus); and validated instruments that ask about use, access and attitudes toward reproductive and sexual health products and services including pregnancy tests, emergency contraception pills, and medication abortion. The survey participants reported previous and preferred locations for use of products and services at five types of healthcare settings; knowledge of whether they could access this healthcare in their community; and sexual and reproductive healthcare behaviors including preventing STIs and pregnancy as applicable. Our assessment of student self-reported use and location knowledge of sexual and reproductive health products and services included barriers to this healthcare overall, and in the context of accessing on-campus student health services, across demographic characteristics.

#### Barriers to care

The study sought to understand the types and extent of barriers students face in accessing reproductive care. We asked participants to report which barriers make it difficult for them or their partner(s) to access reproductive health products and services generally. They were then asked which issues (barriers) have made, or would make, it difficult to access care at the SHC at their university. Both questions provided a multi-option list; respondents could check off as many answers as they wanted. An “Other: Please specify” response option was available.

**Fig. 1:**
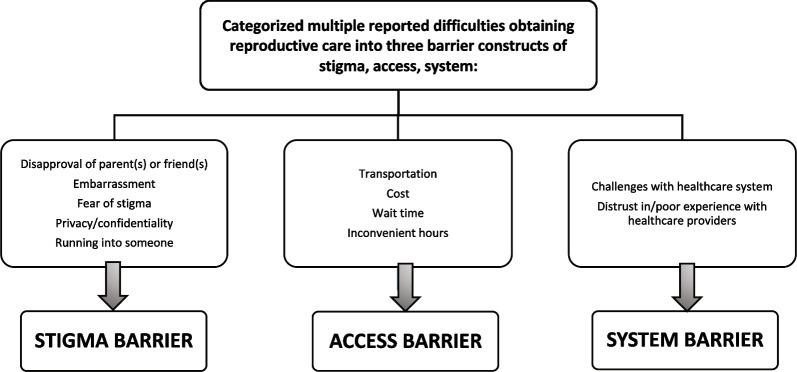
Categorization of barriers to obtaining reproductive & on campus healthcare

To analyze types of barriers experienced generally and at the SHC across the sample population and by demographic subgroups, we categorized question response options into three constructs: (a) Stigma, (b) Access, and (c) System Barriers. Figure 1 details the response options included in each construct.

#### Knowledge of reproductive & sexual healthcare availability

We asked respondents if they knew at least one location in their community where a series of reproductive and sexual health products and services are available. We focused on three services: (1) pregnancy test, (2) emergency contraception pills, and (3) medication abortion. These were selected to reflect an increasingly complex stepwise progression of care for those respondents with the potential for themselves or their partner(s) to become pregnant. A pregnancy test is a product than anyone can purchase without appointment or consultation. Emergency contraception pills are available for over-the-counter (OTC) purchase but require a limited appointment for those needing advice, informational instructions to receive the most effective option for those who weigh over 195 pounds, or payment support through insurance or subsidy program. Lastly, the current U.S. Food and Drug Administration (FDA) and legislation-mandated care model for medication abortion provision requires the highest level of scheduling, clinical supervision and intervention from the healthcare system [17].

#### Attitudes toward use of SHC for reproductive healthcare

We asked respondents if as a student they access the SHC at their university and if they would go to the SHC if they or their partner(s) needed a series of reproductive health products and services. We asked respondents if they would recommend using the SHC to a friend who needed these products and/or services. We focused our analysis on pregnancy test, emergency contraception pills (ex: “morning after pill,” “Plan B,” or “Ella”), and medication abortion.

#### Demographic group measures

To better understand the extent to which certain barriers were most relevant to specific populations and for which populations further outreach and support may be required to address inequities, we categorized responses to be as inclusive as possible. For gender identity, we created a three-category variable of female, male, and transgender/gender expansive (trans/GE). Respondents were categorized as trans/GE who selected: Transgender (n = 2); Nonbinary (n = 9); and Other—Please specify (“Genderqueer” [n = 1]; “He/They” [n = 1]). For race and ethnicity, we allowed respondents to choose from several standard groups (African American, Asian, Pacific Islander, Latino/a, white, mixed race) and also to self-identify into an “other” category. We examined race/ethnicity individually where we had sufficient data, but there were too few Pacific Islanders and “other” respondents to analyze these groups independently. To include respondents across all demographic groups in the analysis, we also further collapsed self-reported racialized and ethnic identities into a three-category variable including (a) underrepresented minority students (URM; African American, Latinx and Pacific Islander), (b) non-underrepresented minority students (non-URM; White and Asian American) and (c) Mixed race/ethnicity students.

### Statistical analyses

We report survey means and 95% confidence intervals to compare barrier types by setting, general (community) reproductive healthcare versus SHC. We examine chi-square tests to analyze differences in barriers by demographic groups. To compare knowledge of where to get services and whether respondents would use or recommend the SHC for each service, we present mean and 95% confidence intervals for each service. We used STATA17 statistical software for all analyses [18].

### Institutional review board approval statement

The University Institutional Review Board provided ethics approval for this study (Protocol 2020-106).

## Results

There were 335 survey responses where respondents engaged with the survey beyond opening the link to preview the questions. We excluded any survey response that was not at least half completed (n = 51); did not consent to be in the study (n = 10); or did not respond to being 18 + years old and attending a California State University (n = 1)*.* The final sample size was n = 273, with 77% of respondents having accessed the survey via a live link distributed with virtual method(s), and 23% via a QR code on the paper flyers.

We compared the demographic characteristic sample means to the study site student population, and our sample was overrepresented by those with female gender identity (72.79%), who are often the focus of reproductive health and abortion services. Transgender, nonbinary and other gender expansive students comprised 4.76% of the sample. Asian American (35.66%), Mixed-race/ethnicity (9.93%) and White (20.22%) students were overrepresented, while African American (3.31%) and Latinx (26.47%) students were underrepresented (Table 1). Students living within 20 miles of campus (72.53%) were overrepresented; and an additional 17.22% of the sample lived approximately within the greater metropolitan area (21–50 miles), and 10.26% greater than 50 miles away.

**Table 1:** Sample characteristics

Variable	Sample		Study Site Population
	n	%	%
Race/ethnicity	273		
African American	9	3.31	5.63
Asian American	97	35.66	27.17
Latinx	72	26.47	36.75
Pacific islander	3	1.10	0.64
Mixed race/ethnicity	27	9.93	5.16
White	55	20.22	17.68
Other^a^	9	3.30	*
Gender identity	272		
Female	198	72.79	56.40
Male	61	22.43	43.31
Trans/gender expansive (GE)^b^	13	4.78	*
Age	271		
18–24 years	200	73.80	*
25–34 years	59	21.77	*
35–44 years	10	3.69	*
45–54 years	2	0.74	*
Anticipated degree	269		
Graduate	35	13.01	15.00
Undergraduate	234	86.99	85.00
Employment status	259		
Full-time	34	13.13	*
Part-time	116	44.79	*
Unemployed	109	42.08	*
Housing situation	266		
On campus	57	21.43	*
Off campus (alone/roommate/partner)	104	39.10	*
Off campus (parent/relatives)	103	38.72	*
Unhoused	2	0.75	*
Distance from campus	273		
0–5 miles	116	42.49	*
5–20 miles	82	30.04	*
20–50 miles	47	17.22	*
> 50 miles	28	10.26	*
Student health center use	267		
Yes	133	49.81	*

^a^: Other—Please specify [Middle Eastern (n = 3); North African (n = 1); Black African (n = 1); Central Asian (n = 1); Samoan/Mexican (n = 1); European (n = 1)]

^b^: Transgender (n = 2); Nonbinary (n = 9); Other—Please specify [Genderqueer (n = 1); “He/They” (n = 1)]

*: No data available for variable or not all categories available in study site population

Most respondents experienced stigma barriers which both made accessing general reproductive healthcare for themselves or their partners difficult to obtain (54.58%; *95% CI* 0.485–0.606), and made accessing the SHC difficult (49.82%; *95% CI* 0.437–0.559). Notably, while the majority of respondents experienced access barriers in both settings, they did so significantly more frequently in general reproductive healthcare (67.77%; *95% CI* 0.619–0.441) than at the SHC (50.18%; *95% CI* 0.441–0.563). Respondents also experienced system barriers significantly more frequently in obtaining general reproductive healthcare (35.16%; *95% CI* 0.295–0.411) than accessing care at the SHC (24.18%; *95% CI* 0.192–0.297).

No statistically significant differences in the three barriers were seen in the sample across racialized/ethnic identities when categorized in two ways; neither by five subgroups of African American, Asian American, Latinx, Mixed race/ethnicity and White; nor when analyzed across groups of non-URM, URM, and Mixed race/ethnicity. Across gender identities, there was a difference in the proportion of system barriers experienced in general reproductive care. Trans/GE identified respondents experienced a significantly higher proportion (84.62%) of system barriers in general reproductive care settings than female (37.88%) and male (16.39%) identified respondents.

Across groups anticipating a graduate and undergraduate degree, a significantly lower proportion of stigma barriers were felt by graduate than undergraduate students in both the general reproductive care and SHC settings. Across subgroups of students living varying distances from campus, students experienced access barriers significantly more frequently overall in reproductive healthcare than at the SHC. In particular, those living inside the local city area (5–20 miles) had less barriers than those living on campus and those living outside the central urban area (> 20 miles) (Table 2).

**Table 2:** Barriers in reproductive health across sample characteristics and setting

Barrier	Stigma						Access***						System**					
Setting	General Repro care			SHC			General Repro care			SHC			General Repro care			SHC		
	%	95% CI		%	95% CI		%	95% CI		%	95% CI		%	95% CI		%	95% CI	
*Panel A. Sample population barriers in general reproductive healthcare and in accessing student health center (SHC)*																		
Sample (n = 273)	54.58	0.485	0.606	49.82	0.437	0.559	67.77	0.619	0.733	50.18	0.441	0.563	35.16	0.295	0.411	24.18	0.192	0.297

Variable	Stigma				Access				System			
	General Repro care		SHC		General Repro care		SHC		General Repro care		SHC	
	%	*p*-value	%	*p*-value	%	*p*-value	%	*p*-value	%	*p*-value	%	*p*-value
*Panel B. reproductive health barriers across characteristics*												
Race/ethnicity		0.378		0.127		0.188		0.297		0.114		0.462
African Amer.	55.56		66.67		100.00		44.44		22.22		44.44	
Asian Amer.	60.82		54.64		62.89		51.55		32.99		23.71	
Latinx	55.56		51.39		70.83		58.33		30.56		19.44	
Mixed Race/Ethn.	55.56		51.85		74.07		37.04		33.33		25.93	
White	43.64		34.55		69.09		43.64		50.91		29.09	
		0.932		0.643		0.397		0.212		0.317		0.864
Non-URM	54.61		47.37		65.13		48.68		39.47		25.66	
URM	57.14		53.57		72.62		55.95		29.76		22.62	
Mixed race/Ethn.	55.56		51.85		74.07		37.04		33.33		25.93	
Gender identity		0.912		0.368		0.116		0.327		0.000		0.462
Female	55.56		52.53		70.71		48.48		37.88		23.23	
Male	52.46		44.26		57.38		52.46		16.39		24.59	
Trans/gender expansive (GE)	53.85		38.46		76.92		69.23		84.62		38.46	
Anticipated degree		0.029		0.001		0.125		0.353		0.534		0.818
Graduate	37.14		22.86		57.14		42.86		40.00		25.71	
Undergrad	56.84		53.85		70.09		51.28		34.62		23.93	
Distance from campus		0.416		0.977		0.005		0.009		0.511		0.796
0–5 miles	58.62		48.28		74.14		54.31		39.66		22.41	
5–20 miles	57.45		51.22		52.44		35.37		29.27		26.83	
20–50 miles	51.22		51.06		72.34		63.83		34.04		21.28	
> 50 miles	42.86		50.00		78.57		53.57		35.71		28.57	

Across reproductive care service types, half to the majority of respondents knew where pregnancy tests, emergency contraception pills and/or medication abortion care was available to them in their community. This proportion was higher for pregnancy test (85.49%; *95% CI* 0.806–0.896) and emergency contraception pills (79.77%; *95% CI* 0.743–0.845) than for medication abortion (49.79%; *95% CI* 0.433–0.563). The willingness for self or partner(s) to use and/or recommend their friend to use care on campus varied by service type. Respondents were less willing to use or recommend medication abortion (60.24%; *95% CI* 0.539–0.663) than for pregnancy test (72.93%; *95% CI* 0.672–0.782) and emergency contraception pills (72.20%; *95% CI* 0.663–0.776) at campus SHC (Table 3).

**Table 3:** Knowledge and use of reproductive services in community and at student health Center (SHC)

	Knowledge of where services available in community				Would use/partner(s) use, or recommend friend to use, at SHC			
Service	n	%	95% CI		n	%	95% CI	
Pregnancy test	255	85.49	0.806	0.896	266	72.93	0.672	0.782
Emergency Contraception pills	257	79.77	0.743	0.845	259	72.20	0.663	0.776
Medication abortion	239	49.79	0.433	0.563	254	60.24	0.539	0.663

## Discussion

The SHC is a trusted and known source of reproductive health products and services, with a significantly lower proportion of access and system barriers for students than in general reproductive healthcare. SHCs are thus essential and accessible settings which can be leaders in lowering reproductive and abortion care barriers for the large population of increasingly diverse college students. Our research of student experience and attitudes supports the appropriateness of legislation turning to SHCs to increase reproductive health access, including medication abortion at public universities. However, while students report high willingness to use and recommend the SHC, they are less likely to do so for medication abortion than a pregnancy test or emergency contraception pills. This may reflect the fact that medication abortion is not yet available at the SHC. However, it also reveals a critical gap and need as California public university SHCs initiate the provision of this essential care. Consideration and prioritization of how to ensure equitable delivery of this safe, highly effective, cost-efficient, increasingly common yet increasingly restricted service must be continually informed by research centering students’ voices and the experiences of people with barriers to abortion access [19, 20].

Our research has multiple implications for university health education and SHC stakeholders in their role reducing reproductive health and abortion care barriers across student populations, and in better serving student needs. Students have high levels of knowledge about where to access most reproductive healthcare products and services and are willing to utilize SHCs for this care. However, SHCs must account for the majority proportion of students experiencing stigma, access, and system barriers even in a large coastal city with state legislation supportive of reproductive and abortion access [2, 17]. By continuing to document who has knowledge, trust, and barriers while implementing new services, SHCs can monitor gaps and successes, and maintain focus on communities of need with culturally relevant care to diverse student populations. Outreach and support are critical, for example, to decrease stigma barriers for the majority of undergraduate students who experience them, and to engage the  majority of trans/GE students who experience system barriers in general care settings [21].

Our research strengths include focus on student voice and experience in building on previous work identifying the important roles of SHCs. We rely on students as expert informants of their own health care needs and autonomous care decisions. The student perspective presented here also reflects students embedded in a diverse urban population, including a significant proportion of trans/GE students who experience notable barriers across and among demographic characteristics. We document student barriers despite perceptions of this setting as one with high levels of access to reproductive care including abortion. While previous research shows average medication abortion access (travel distance and soonest appointment availability) and lower than average cost at our study location compared to other California public universities, the majority of respondents (68%) experience difficulties accessing reproductive care [3]. Our documentation of baseline knowledge of service locations, attitudes toward accessing the SHC, and experienced barriers can strongly inform rollout of increased levels of care in California and elsewhere.

Limitations in the study include our recruitment methods’ potential influence on the sample population, as seen in the overrepresented proportion of students living on campus as well as the modest response rate relative to the target population. An additional limitation is inclusion of only one California public university site, and further research including larger sample sizes may be necessary to confirm findings of a lack of significant barrier differences across racialized/ethnic identities in the sample. There may be specific barriers related to provision of reproductive and medication abortion care in an SHC setting that we did not represent or probe due to utilizing existing validated survey instruments [1, 7]. Future work can be informed by dialog with students, SHCs, and community reproductive health and medication abortion providers, staff, and organizers to document any important domains missed here.

Continuation of this research at other California public university sites outside of this Northern California coastal urban area is critical to evaluating barriers and successes and enhancing wellbeing for students across regions and urbanicities. As seen in multiple components included in the assessed stigma barriers (*concerns for privacy and confidentiality, fear of disapproval from parents or friends*), students living with relatives or roommates and others without a private and safer space to experience bleeding and cramping during medication abortion (such as the unhoused) may be further at risk for inequities in access, care and outcomes. Thus, implications of student housing situation are particularly relevant to medication abortion, and other living situations than distance from campus should be addressed in future research. Current California legislation SB-24 includes SHC funding, coverage of cost of services, and medical support to SHCs but was limited to four-year public institutions, leaving a considerable gap for other students. Community colleges notably have higher proportions of low SES, URM, and parenting students coupled with fewer (or lack of) SHC services, thus it is critical that future student health equity research and policy includes these students and settings [22].

Medication abortion is the most prevalent and growing method of abortion and is increasingly done without an in-person visit both within (telemedicine) and outside the confines of the formal healthcare system (self-management) [20]. International practice, user comfort, and research increasingly shows evidence for this service as an “ideal candidate” for over-the-counter use with the potential to reduce barriers and increase reproductive autonomy [17, 23]. Our findings of decreased student willingness to seek medication abortion at the SHC as compared to other health products relevant to telemedicine and OTC provision can inform further research into student perspectives and SHC challenges and successes in telemedicine and OTC reproductive health models. The recent ruling by the FDA allowing pharmacies to dispense Mifepristone suggests that they are endorsing telemedicine and OTC models, which are safe and effective modes to increase access, health equity, and patient-centered abortion care [2, 5, 17, 24].

In a nation where the constitutional right to abortion is no longer protected, states choosing to guarantee and expand reproductive services including abortion care can partner with and support SHCs. SHCs are trusted, accessible, and have a unique public health role in increasing health, academic and gender equity for the sizeable population of college students. Thus, SHCs are well positioned to support students. In particular, where state laws permit, university health centers can support students' reproductive health options by providing medication abortion. Where prohibited by state laws, they can still provide neutral and factual information about the laws and options as a trusted source of information for students [25]. More attention and financial support must be paid to university health centers as they are being called upon to lead an expansion of reproductive and abortion care.

## Data Availability

The datasets generated and analyzed during the current study are not publicly available due to continuation of the survey in future years but are available from the corresponding author on reasonable request. When the data collection is complete, the data will be deposited into the Mendeley data repository.

## Abbreviations

BIPOC: Black, indigenous, people of color
CI: Confidence interval
Non-URM: Non-underrepresented minority
OTC: Over-the-counter
SES: Socioeconomic status
SHC(s): Student health center(s)
STIs: Sexually transmitted infections
Trans/GE: Transgender and/or gender expansive
URM: Underrepresented minority
